# Altered Ceramide Profile of Facial Sensitive Skin: Disordered Intercellular Lipid Structure Is Linked to Skin Hypersensitivity

**DOI:** 10.1111/jocd.70154

**Published:** 2025-04-02

**Authors:** Taisei Joichi, Hiroyuki Yoshida, Hiroaki Katsukura, Lili Zhai, Daisuke Watanabe, Naohito Yamamoto, Mai Haneoka, Shun Nakamura, Akane Kawamoto, Hiromitsu Nakazawa, Motoaki Suka

**Affiliations:** ^1^ Skin Care Products Research Kao Corporation Odawara Kanagawa Japan; ^2^ Biological Science Research Kao Corporation Odawara Kanagawa Japan; ^3^ Analytical Science Research Kao Corporation Tochigi Japan; ^4^ School of Science Kwansei Gakuin University Sanda Hyogo Japan

**Keywords:** ceramides, lipid, sensitive skin

## Abstract

**Background:**

Although sensitive skin (SS) is a syndrome characterized by cutaneous hypersensitivity to environmental factors, its pathophysiology remains elusive.

**Aims:**

We aimed to explore the characteristics of ceramides (CERs) and intercellular lipid (ICL) structures of individuals with and without facial SS and their relationship with skin hypersensitivity.

**Patients/Methods:**

Healthy Japanese females were divided into SS or non‐SS groups based on self‐perception and lactic acid stinging test (LAST). Stratum corneum (SC) lipids were analyzed using a liquid chromatograph‐mass spectrometer, and the orthorhombic‐hexagonal lateral packing structure of ICLs was assessed using electron diffraction.

**Results:**

According to the mean LAST score, individuals with SS (*n* = 48) had mild‐to‐moderate skin hypersensitivity. SS exhibited not significantly but slightly impaired skin barrier function (*p* = 0.072) and lower levels of CER[NH], [NP], [EOS], [EOH] (all *p* < 0.05), and [EOP] (*p* = 0.073) in the SC compared with non‐SS (*n* = 18). Notably, the CER[NP]/[NS] ratio, a marker of skin barrier function, was positively correlated with the orthorhombic‐hexagonal lateral packing ratio of ICLs (*p* = 0.002), whereas it was negatively correlated with the LAST score (*p* = 0.015) and the interleukin (IL)‐1 receptor antagonist/IL‐1α ratio (*p* = 0.003) in the SC, an indicator of chronic inflammation. Moreover, corneocyte size was reduced in SS (*p* < 0.001), suggesting inferior SC maturation, and was positively correlated with the CER[NP]/[NS] (*p* < 0.001) and the orthorhombic‐hexagonal ratios (*p* = 0.011).

**Conclusions:**

Individuals with SS showed an abnormal CER profile, particularly the altered CER[NP]/[NS] ratio, which was in turn associated with disordered ICL structure and skin hypersensitivity. Abnormal epidermal turnover may be an underlying mechanism of the abnormalities.

**Abbreviations**
ad
: atopic dermatitis
APCI: atmospheric pressure chemical ionization
CER: ceramide
CER[ADS]: α‐hydroxy fatty acid/dihydrosphingosine base ceramide
CER[AH]: α‐hydroxy fatty acid/6‐hydroxy‐sphingosine base ceramide
CER[AP]: α‐hydroxy fatty acid/phytosphingosine base ceramide
CER[AS]: α‐hydroxy fatty acid/sphingosine base ceramide
CER[EOH]: ester‐linked ω‐hydroxy fatty acid/6‐hydroxy‐sphingosine base ceramide
CER[EOP]: ester‐linked ω‐hydroxy fatty acid/phytosphingosine base ceramide
CER[EOS]: ester‐linked ω‐hydroxy fatty acid/sphingosine base ceramide
CER[NDS]: non‐hydroxy fatty acid/dihydrosphingosine base ceramide
CER[NH]: non‐hydroxy fatty acid/6‐hydroxy‐sphingosine base ceramide
CER[NP]: non‐hydroxy fatty acid/phytosphingosine base ceramide
CER[NS]: non‐hydroxy fatty acid/sphingosine base ceramide
CHOL: cholesterol
DES: dihydroceramide desaturase
EAD: extrinsic atopic dermatitis
EI: erythema index
ESI: electrospray ionization
FFA: free fatty acid
IAD: intrinsic atopic dermatitis
ICL: intercellular lipid
IgE: immunoglobulin E
IL‐1ra: interleukin‐1 receptor antagonist
IL‐1α: interleukin‐1α
LAST: lactic acid stinging test
LC–MS: liquid chromatograph‐mass spectrometer
SC: stratum corneum
SIM: selected ion monitoring
SS: sensitive skin
TARC: thymus and activation‐regulated chemokine
TEWL: trans‐epidermal water loss

## 1 Introduction

Sensitive skin (SS) is a syndrome defined by the occurrence of unpleasant sensations (stinging, burning, pain, pruritus, and tingling sensations) in response to stimuli that normally should not provoke such sensations. SS can affect all body parts, especially the face, and affects approximately 50% of the world's population based on self‐assessment questionnaires [1]. Although the pathophysiology of SS is not fully understood, hypersensitivity of the cutaneous neuronal system and/or abnormal skin barrier function, which may be associated with smaller and immature corneocytes, have been hypothesized to be the primary culprit [1, 2].

Intercellular lipids (ICLs) in the stratum corneum (SC) play an essential role in the permeability barrier function by preventing water loss and penetration of external irritants. ICLs comprise ceramides (CERs), free fatty acids (FFAs), and cholesterol (CHOL), with CERs as the major component. SS is more common in patients with atopic dermatitis (ad) and psoriasis than in healthy individuals, and the levels of CERs in those patients are lower than those in healthy controls [3, 4]. Thus, the CER levels are probably associated with disrupted skin barrier function in SS under such pathological conditions [1]. On the other hand, as for “healthy” SS, only a few conflicting results have been reported regarding the CER levels, particularly of “self‐perceived” SS. For instance, a clinical study reported that the quantity of CERs in self‐perceived SS was significantly lower than that in self‐perceived non‐SS, despite no significant difference in the facial skin barrier measured by trans‐epidermal water loss (TEWL) and sensation evaluated by a 10% lactic acid stinging test (LAST) for 10 min between them [5]. Another study showed that the levels of total CERs, as well as SC thickness, water, and natural moisturizing factors, were not significantly different between the self‐perceived SS and non‐SS groups [6]. One possible reason for the conflicting results may be derived from the perception‐based subjective evaluation to categorize SS, which can potentially include SS mainly associated with psychological factors, including negative emotions and stress that may contribute to the development of itch sensations. Moreover, changes in the composition of 12 CER subclasses and an increase in short‐chain CERs have been reported to be correlated with disrupted barrier function and altered lipid organization in ad patients [3, 7]. Nevertheless, a direct link between skin hypersensitivity and CER profile or chain length distribution in the SC remains to be investigated.

Collectively, no direct evidence exists regarding the role of CERs in cutaneous barrier mechanism(s) and hypersensitivity in healthy individuals with SS. In this study, we aimed to investigate the relationship between the facial skin properties and CERs of individuals with SS and those without SS categorized by using a self‐perception‐based questionnaire and 1% LAST.

## 2 Materials and Methods

### 2.1 Study Participants

The healthy Japanese females (age range, 20–49 years) matching the inclusion criteria were recruited for this study. Based on their responses to the self‐assessment questionnaire related to facial skin sensitivity, the individuals were enrolled in the self‐perceived SS group or the self‐perceived non‐SS group. LAST was performed as described below, and the individuals (LAST score ≥ 1) in the self‐perceived SS group were classified as individuals with SS (mean age ± standard deviation [SD], 36.52 ± 8.63 years) and the individuals (LAST score < 1) in the self‐perceived non‐SS group were classified as individuals without SS (mean age ± SD, 35.11 ± 10.49 years). To avoid a case where the self‐perceived SS group is mainly occupied by individuals who are associated with psychological factors, not skin physiological factors, a greater number of individuals in the self‐perceived SS group were recruited than those in the self‐perceived non‐SS group. Individuals with skin diseases, including ad and ichthyosis vulgaris, severe allergies, those who are pregnant, or those who may be lactating, were excluded. Prior to the measurement of facial skin physiological parameters, all individuals washed their faces and acclimated in a room with constant temperature (21.5°C ± 1.0°C) and humidity (49.5% ± 4.5%) for 15 min. This study was conducted in accordance with the Declaration of Helsinki and was approved by the ethics committee of Kao Corporation (study number: D164‐210115). Informed consent was obtained from all participants.

### 2.2 LAST

LAST was performed with a 20 mm × 50 mm square piece of cellulose nonwoven cloth soaked with 400 μL distilled water or aqueous solution containing 1% lactic acid heated to 32°C. Distilled water was applied by placing the cloth on the left side of the cheek. The individuals reported the intensity of the sensation as pain, burning, itch, or crawly feeling using a 4‐point scale (severe = 3; moderate = 2; mild = 1; non = 0) at 0.5, 2.5, and 5 min after application. Half‐intermediate scores (every 0.5) were used for the evaluation. After a 5 min interval, the 1% lactic acid solution was applied to the same skin area, and the individuals reported the intensity of the sensation. The LAST score was calculated using a previously proposed formula [8]. Briefly, the maximum score from the three time points for each type of sensation was analyzed. The difference in one of the maximum scores between 1% lactic acid and distilled water was calculated and defined as the LAST score.

### 2.3 Measurement of TEWL and Skin Hydration (Capacitance)

TEWL (gm^−2^ h^−1^) and skin hydration (A.U.) were measured on the right side of the cheek using the Tewameter TM300 (Courage+Khazaka electronic, Cologne, Germany) and the Corneometer CM825 (Courage+Khazaka electronic, Cologne, Germany), respectively.

### 2.4 Measurements of Skin Redness Parameters

The a* and spectral reflectance values were measured on the right side of the cheek using a CM2600d (Konica Minolta, Tokyo, Japan). Erythema index (EI) was calculated using spectral reflectance and a previously proposed formula [9].

### 2.5 Analysis of Serum

Blood samples were taken: 6 mL for biochemical test and 2 mL for hematology test. Samples for the biochemical test were allowed to clot for 40 min at room temperature, then centrifuged at 15000 *g* for 15 min. Serum was collected, and the levels of lactate dehydrogenase, nonspecific immunoglobulin E (total IgE), and thymus and activation‐regulated chemokine (TARC) were measured using LABOSPECT 008 K (Hitachi High‐Tech, Tokyo, Japan) or BioMajesty Series JCA‐BM8060 (JEOL, Tokyo, Japan), an immunofluorescence analyzer Phadia5000 (Thermo Fisher Scientific, MA, USA), and a fully automated immunoassay system HISCL‐5000 (Sysmex, Hyogo, Japan), respectively. Samples for hematology test were refrigerated at 4°C after blood collection, and the number of white blood cells, basophils, eosinophils, lymphocytes, monocytes, and neutrophils was counted using an automated hematology analyzer XN‐10 (Sysmex, Hyogo, Japan).

### 2.6 Liquid Chromatograph–Mass Spectrometer (LC–MS) Analysis of ICLs in Tape‐Stripped SC

SC samples were collected by tape‐stripping from the right side of the cheek using 25 mm × 30 mm square pieces of film‐masking tape 465 #40 (Teraoka Seisakusho, Tokyo, Japan). Three consecutive tape strips were used for each participant. The ICLs were extracted from half of each tape sample with 1.8 mL of methanol/2‐propanol/chloroform (9:9:2, v/v/v). The LC–MS analysis of ICLs was performed as previously [10] using an Agilent 6130 Series LC/MSD SL system equipped with an Agilent 1260 Infinity Series LC, multi‐ion source, and ChemStation software (Agilent Technologies, Santa Clara, CA, USA). The sample solution was a mixture of 180 μL of the ICL extraction and 20 μL of internal standard solution (500 nM of N‐heptadecanoyl‐D‐erythro‐sphingosine [Avanti Polar Lipids, Alabaster, AL, USA], 10 μM of arachidic acid‐d39 [Cambridge Isotope Laboratories, Tewksbury, MA, USA], 10 μM of CHOL‐d7 [Avanti Polar Lipids], and 2.5 μM sodium icosyl sulfate [Santa Cruz Biotechnology, Dallas, TX, USA] in methanol/2‐propanol/chloroform [9:9:2, v/v/v]). CERs, FFAs, and CHOL sulfate were detected by selected ion monitoring (SIM) of electrospray ionization (ESI) mode as m/z [M‐H]^−^, and free CHOL was detected by SIM of atmospheric pressure chemical ionization (APCI) mode as m/z [M + H‐H_2_O]^+^. Total SC protein level of half of each tape sample was quantified as previously [11] using a BCA kit (Thermo Scientific, Rockford, IL, USA), and the levels of CER subclasses, FFAs, free CHOL, and CHOL sulfate were normalized by quantitated protein level.

### 2.7 Analysis of the Lateral Packing Structure of ICLs

SC samples were collected from the right side of the cheek using the grid‐stripping method, and the lateral packing structure of ICLs was analyzed using low‐flux electron diffraction [12, 13]. Corneocytes that adhered to the grid were applied to a conventional transmission electron microscope (JEM1400, JEOL) operated at an acceleration voltage of 100 kV. Electron diffraction images of randomly selected areas of 55 μm^2^ were obtained from at least 24 corneocytes collected evenly from within a 3 cm × 3 cm area at each site of each participant, excluding areas where corneocytes overlapped. The electron dose for an image was approximately 1.4 e∙nm^−2^, which is small enough not to damage the structural organization of ICLs according to a previous study [14]. For quantitative analysis, we calculated electron diffraction intensity profiles as a function of the scattering vector (*s* = 2sin*θ*/*λ*) by integrating the intensity along the azimuthal direction to achieve high‐resolution analysis by not considering the anisotropy of the diffraction patterns. The peak intensity ratio (Pk2.7/Pk2.4) of the diffraction peaks at *s* ≈ 2.4 nm^−1^ and *s* ≈ 2.7 nm,^−1^ derived from hexagonal and orthorhombic packing structures, was evaluated to clarify the difference in packing structure of ICLs between non‐SS and SS [12].

### 2.8 Measurement of Corneocyte Size

Tape‐stripping was performed on the right side of the cheek using a CAF sheet (Toppan, Tokyo, Japan). The stripped SC sheets were immersed in an aqueous solution containing 0.5% brilliant green (FUJIFILM Wako Pure Chemical, Osaka, Japan) and 1% gentian violet (FUJIFILM Wako Pure Chemical) for 10 min. After washing with distilled water, the sheets were completely dried and sealed on a glass slide with Malinol 750cps (MUTO PURE CHEMICALS, Tokyo, Japan). Corneocyte size was analyzed using images captured via microscopy with corneocytometry 2.0 (CIEL, Osaka, Japan).

### 2.9 Determination of Interleukin‐1 Receptor Antagonist (IL‐1ra) and IL‐1α Levels

Tape stripping was performed on the right side of the cheek using 25 mm × 30 mm square pieces of film‐masking tape 465#40 (Teraoka Seisakusho, Tokyo, Japan). Three consecutive tape strips were employed for each participant. Proteins were extracted from the SC tapes via shaking in Dulbecco's phosphate‐buffered saline containing 0.1% Triton X‐100 overnight at 5°C. After sonication for 30 min under ice‐cold conditions, the solution was centrifuged at 15 000 *g* for 30 min at 4°C, and the supernatants were collected as SC extracts. Total SC protein levels were quantified using a Micro BCA Protein Assay Kit (Thermo Fisher Scientific, Waltham, MA, USA), and IL‐1ra and IL‐1α levels were determined using a Quantikine ELISA Kit (R&D Systems, Minneapolis, MN, USA).

### 2.10 Statistical Analysis

Statistical significance was assessed using the Mann–Whitney *U* test, Student's *t* test, or Welch's *t* test, and correlations were examined using Pearson's or Spearman's correlation analysis with Excel (Microsoft 365) (Microsoft, Redmond, WA, USA) or IBM SPSS Statistics 28.0 (IBM, Armonk, NY, USA). Nonparametric statistics (Mann–Whitney *U* test and Spearman's correlation analysis) and parametric statistics (Student's *t* test, Welch's *t* test, and Pearson's correlation analysis) were used for the data measured on an ordinal scale of measurement and ratio scales of measurement, respectively. Welch's *t* test was used for total IgE because the data had unequal variances. A *p* value < 0.05 was considered to indicate statistical significance.

## 3 Results

### 3.1 Characteristics of Study Individuals With and Without SS

**FIGURE 1:**
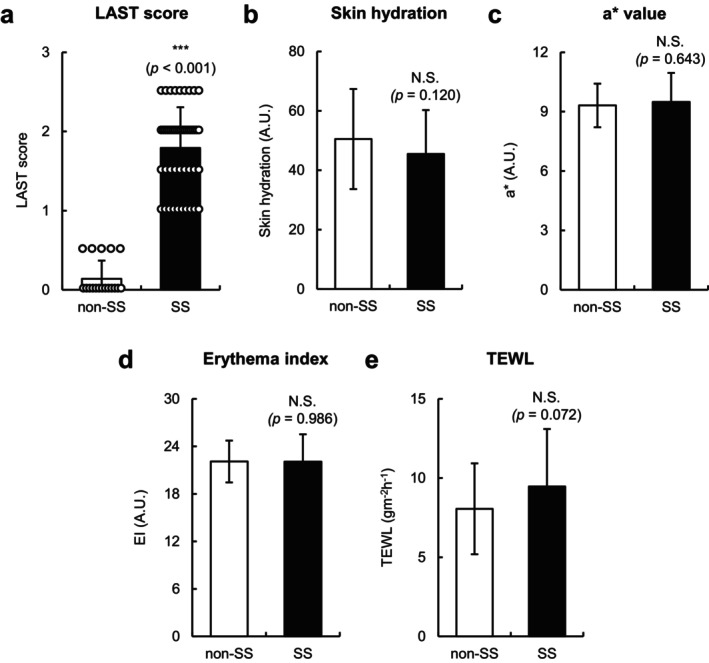
Skin hypersensitivity and physiological properties of individuals with and without sensitive skin (SS). (a**–**e) Scores of 1% lactic acid stinging test (LAST) (a), skin hydration values (b), a* values (c), erythema index (EI) (d), and trans‐epidermal water loss (TEWL) values (e) of individuals without SS (non‐SS) (*n* = 18) and those with SS (*n* = 48). Values are expressed as mean ± SD (standard deviation). Statistical significances were assessed using Mann–Whitney *U* test for (a) or Student's *t* test for (b–e). ***p < 0.001. N.S., not significant. A.U., arbitrary unit.

The LAST score of individuals with SS (*n* = 48) and those without SS (*n* = 18) was 1.8 ± 0.5 and 0.1 ± 0.2 (mean ± SD), respectively (Figure 1a), indicating that individuals with SS had mild‐to‐moderate facial skin hypersensitivity. Although no significant differences in facial skin hydration, a* values, and EI values were found between individuals with SS and those without SS (Figure 1b–d), the TEWL of individuals with SS tended to be higher than that of non‐SS (*p* = 0.072) (Figure 1e). Notably, the mean age was not significantly different between the two groups due to the limited age range (20–49 years) in this study (Table 1). In addition, although no significant differences were found in the levels of white blood cells, basophils, eosinophils, lymphocytes, monocytes, neutrophils, lactate dehydrogenase, and TARC in the sera of individuals with SS and those without SS, total IgE levels were modestly but significantly higher in individuals with SS compared with non‐SS (*p* = 0.018) (Table 1). These results suggest that individuals with SS showed no apparent skin or immune abnormalities but exhibited slightly disrupted facial skin barrier function and mildly elevated serum IgE levels.

**TABLE 1:** Age and blood serum composition of individuals with and without sensitive skin (SS).

	non‐SS (mean ± SD)	SS (mean ± SD)	*p*
Age (years)	35.1 ± 10.4	36.5 ± 8.6	0.579
White blood cells (/μL)	6023.9 ± 1271.3	5774.0 ± 1410.1	0.513
Basophils (%)	0.7 ± 0.3	0.9 ± 0.4	0.138
Eosinophils (%)	2.4 ± 2.3	2.7 ± 2.3	0.632
Lymphocytes (%)	35.0 ± 9.3	32.6 ± 6.6	0.261
Monocytes (%)	5.7 ± 0.9	5.8 ± 1.4	0.798
Neutrophils (%)	56.2 ± 9.4	58.0 ± 7.5	0.429
Lactate dehydrogenase (U/L)	150.8 ± 25.0	156.0 ± 22.8	0.422
**Total IgE (IU/L)**	**56.2 ± 47.2**	**127.5 ± 187.1**	**0.018** *
TARC (pg/mL)	256.8 ± 98.0 (*n* = 17)	283.0 ± 129.1	0.450

*Note:* Age and the levels of white blood cells, basophils, eosinophils, lymphocytes, monocytes, neutrophils, lactate dehydrogenase, nonspecific immunoglobulin E (total IgE), and thymus and activation‐regulated chemokine (TARC) in the sera of individuals without SS (non‐SS) (*n* = 18) and those with SS (*n* = 48). Values are expressed as mean ± SD. Statistical significances were assessed using Student's *t* test or Welch's *t* test for total IgE alone. Bold values indicate statistical significance.

Abbreviation: SD, standard deviation.

* *p* < 0.05.

### 3.2 Changes in the CER Profile of Individuals With SS

**FIGURE 2:**
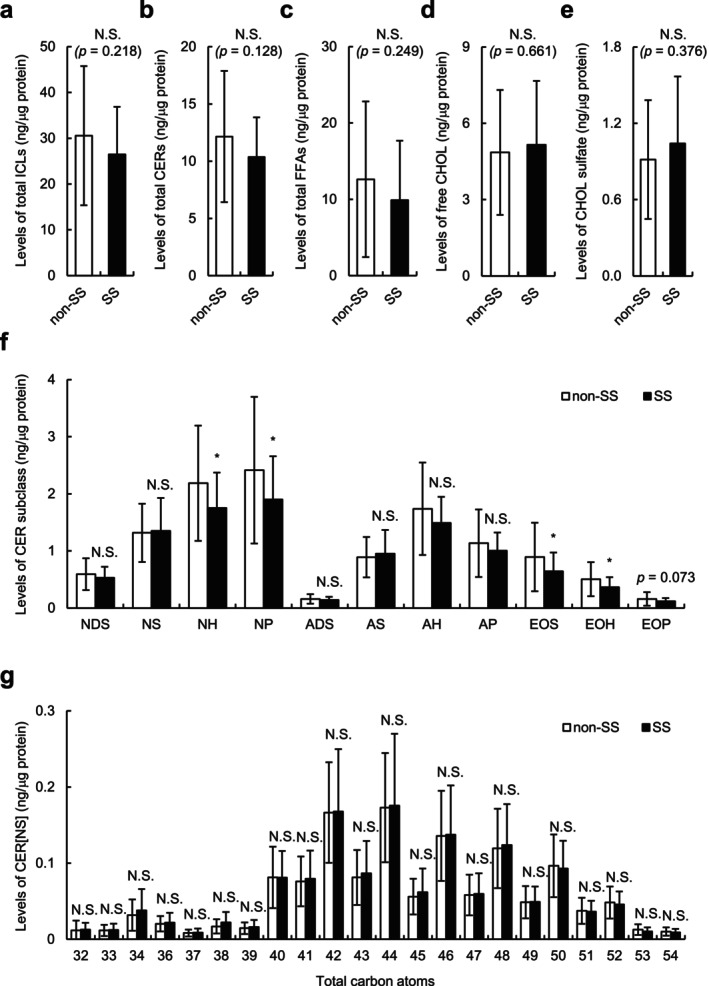
Intercellular lipids (ICLs) in the stratum corneum (SC) of individuals with and without SS. (a**–**d) Levels of total ICLs (a), total ceramides (CERs) (b), total free fatty acids (FFAs) (c), free cholesterol (CHOL) (d), and CHOL sulfate (e) in tape‐stripped SC from individuals without SS (non‐SS) (*n* = 18) and those with SS (*n* = 48). (f) Levels of CER[NDS], [NS], [NH], [NP], [ADS], [AS], [AH], [AP], [EOS], [EOH], and [EOP] in the tape‐stripped SC. (g) Levels of CER[NS] containing 32–54 total carbon atoms in the tape‐stripped SC. Values are expressed as mean ± SD (standard deviation). Statistical significances were assessed using Student's *t* test. *p < 0.05. N.S., not significant.

We examined the SC lipids extracted from tape‐stripped SC samples using an LC–MS. As shown in Figure 2a–e, no significant differences were found in the levels of total ICLs and their major constituents, such as CERs, FFAs, free CHOL, and CHOL sulfate between individuals with SS and those without SS. We then examined the levels and average carbon chain length of the CER subclass in individuals with SS and those without SS. As shown in Figure 2f, the levels of CER[NH], [NP], [EOS], and [EOH] were significantly lower (all *p* < 0.05), and CER[EOP] level tended to be lower in individuals with SS than those without SS (*p* = 0.073). No significant reduction in the average carbon chain length of all CERs (Figure S1), including CER[NS], which is known to be associated with disrupted skin barrier function in ad patients [3] (Figure 2g), was found in individuals with SS compared with those without SS. Similar results were obtained with linear, branched, and unsaturated FFAs (Figure S2).

### 3.3 Relationship Between the Decreased CER[NP]/[NS] Ratio Found in Individuals With SS and Facial Skin Hypersensitivity

**FIGURE 3:**
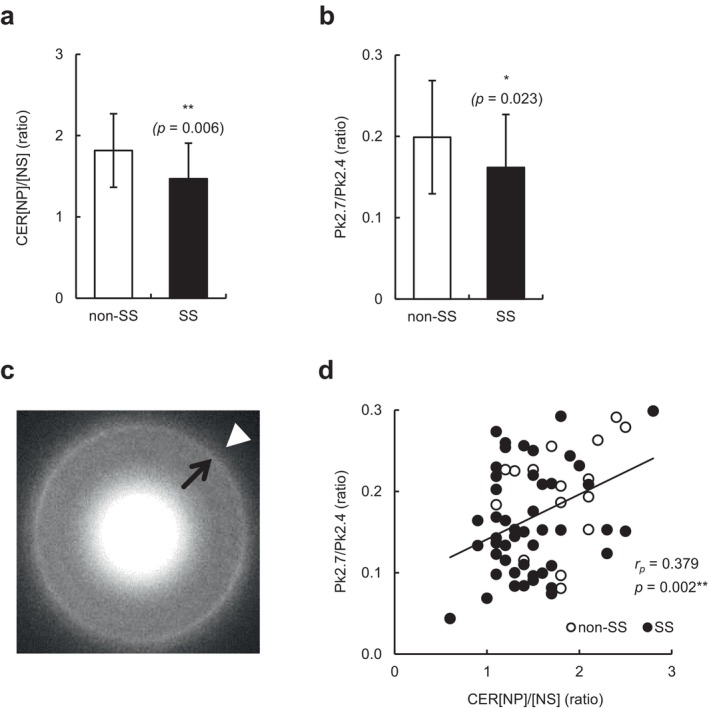
CER[NP]/[NS] and Pk2.7/Pk2.4 ratios in the SC of individuals with and without SS, and correlation between them. (a**–**b) CER[NP]/[NS] ratio (a) and orthorhombic‐hexagonal lateral packing ratio of intercellular lipids (Pk2.7/Pk2.4 ratio) (b) in tape‐stripped SC from individuals without SS (non‐SS) (*n* = 18) and those with SS (*n* = 48). Values are expressed as mean ± SD (standard deviation). Statistical significances were assessed using Student's *t* test. **p < 0.01; *p < 0.05. (c) The representative electron diffraction pattern of corneocytes collected from the cheek. The black arrow and white arrowhead indicate positions of Pk2.4 and Pk2.7, respectively. (d) Correlation between CER[NP]/[NS] and Pk2.7/Pk2.4 ratios in all participants of the two groups (*n* = 66). Open and closed circles indicate non‐SS and SS, respectively. Correlation was examined using Pearson's correlation analysis. *r*_*p*_, Pearson's correlation coefficient. **p < 0.01.

We then examined the relationship between CER levels and skin hypersensitivity on the face by combining data from individuals with and without SS. Consistent with previous observations [3], the levels of CER[NH], [NP], [EOS], [EOH], and [EOP] were significantly correlated with TEWL and/or skin hydration values (all *p* < 0.05) (Table 2); however, the levels of total CERs and any CER subclass did not significantly correlate with the LAST score (Table 2). Thus, we focused on the CER[NP]/[NS] ratio, which is extensively correlated with the functional and biophysical properties of SC [11]. As shown in Figure 3a, the CER[NP]/[NS] ratio was significantly lower in individuals with SS than in those without SS (*p* = 0.006). Moreover, the CER[NP]/[NS] ratio was not only negatively correlated with TEWL values but also with the LAST score (*p* < 0.001 and *p* = 0.015, respectively) (Table 2). To further examine the role of the altered CER profile in the orthorhombic‐hexagonal lateral packing structure of ICLs and facial skin hypersensitivity, we analyzed the Pk2.7/Pk2.4 ratio using grid‐stripped SC and electron diffraction. As shown in Figure 3b,c, individuals with SS had a significantly lower Pk2.7/Pk2.4 ratio than non‐SS (*p* = 0.023). Notably, the levels of CER[NH], [NP], [EOS], [EOH], and [EOP], and the CER[NP]/[NS] ratio were positively correlated with the Pk2.7/Pk2.4 ratio (all *p* < 0.05), and particularly the CER[NP]/[NS] ratio showed the highest correlation among them (*p* = 0.002) (Table 2 and Figure 3d). In addition, the Pk2.7/Pk2.4 ratio, TEWL, and total IgE levels, which were significantly or tended to be different between individuals with SS and those without SS, did not significantly correlate with the LAST score (Table 3). Taken together, the CER[NP]/[NS] ratio is the most greatly related to facial skin hypersensitivity, and it may be linked to slightly impaired skin barrier function via the disordered lateral packing structure of ICLs.

**TABLE 2:** Relationship between ceramide (CER) profile and skin physiological properties associated with sensitive skin.

	TEWL (gm^−2^ h^−1^)		Skin hydration (A.U.)		LAST score		Pk2.7/Pk2.4 (ratio)	
	*r*_*p*_	*p*	*r*_*p*_	*p*	*r*_*s*_	*p*	*r*_*p*_	*p*
**Total CERs**	−0.218	0.079	**0.278**	**0.024** *	−0.113	0.365	0.218	0.079
CER[NDS]	−0.108	0.388	0.239	0.054	−0.085	0.497	0.163	0.191
**CER[NS]**	0.089	0.476	0.136	0.277	−0.021	0.865	−0.054	0.669
**CER[NH]**	**−0.329**	**0.007** **	**0.326**	**0.008** **	−0.159	0.201	**0.333**	**0.006** **
**CER[NP]**	**−0.287**	**0.020** *	**0.278**	**0.024** *	−0.166	0.182	**0.309**	**0.012** *
CER[ADS]	−0.125	0.318	0.237	0.055	−0.051	0.687	0.129	0.300
CER[AS]	0.117	0.348	0.055	0.661	−0.012	0.922	−0.232	0.061
**CER[AH]**	**−0.315**	**0.010** *	**0.315**	**0.010** **	−0.117	0.351	0.186	0.134
CER[AP]	−0.191	0.125	0.192	0.123	−0.130	0.298	0.183	0.142
**CER[EOS]**	**−0.287**	**0.019** *	**0.257**	**0.037** *	−0.179	0.150	**0.365**	**0.003** **
**CER[EOH]**	**−0.328**	**0.007** **	**0.293**	**0.017** *	−0.141	0.260	**0.322**	**0.008** **
**CER[EOP]**	−0.118	0.345	**0.331**	**0.007** **	−0.005	0.965	**0.327**	**0.007** **
**CER[NP]/[NS] (ratio)**	**−0.426**	**< 0.001** ***	0.159	0.203	**−0.299**	**0.015** *	**0.379**	**0.002** **

*Note:* Correlation of total CER levels, CER subclass levels, or CER[NP]/[NS] ratio with trans‐epidermal water loss (TEWL), skin hydration, lactic acid stinging test (LAST) score, or orthorhombic‐hexagonal lateral packing ratio of intercellular lipids (Pk2.7/Pk2.4 ratio) in all participants (*n* = 66). Correlations were determined using Pearson's correlation analysis or Spearman's correlation analysis for LAST score only. Bold values indicate statistical significance.

Abbreviations: A.U., arbitrary unit; *r*_*p*_, Pearson's correlation coefficient; *r*_*s*_, Spearman's correlation coefficient.

*** *p* < 0.001.

** *p* < 0.01.

* *p* < 0.05.

**TABLE 3:** Relationship between skin physiological properties and skin hypersensitivity.

	LAST score	
	*r*_*s*_	*p*
TEWL (gm^−2^ h^−1^)	0.180	0.149
Total IgE (IU/L)	0.075	0.548
Pk2.7/Pk2.4 (ratio)	−0.241	0.051
Corneocyte size (μm^2^)	−0.240	0.053

*Note:* Correlation of trans‐epidermal water loss (TEWL), nonspecific immunoglobulin E (total IgE) levels, orthorhombic‐hexagonal lateral packing ratio of intercellular lipids (Pk2.7/Pk2.4 ratio), or corneocyte size with lactic acid stinging test (LAST) score in all participants (*n* = 66). Correlations were determined using Spearman's correlation analysis.

Abbreviation: *r*_*s*_, Spearman's correlation coefficient.

### 3.4 Possible Contribution of Chronic Subclinical Inflammation to the Abnormal CER[NP]/[NS] Ratio and Lipid Organization via an Increased Epidermal Turnover Rate

**FIGURE 4:**
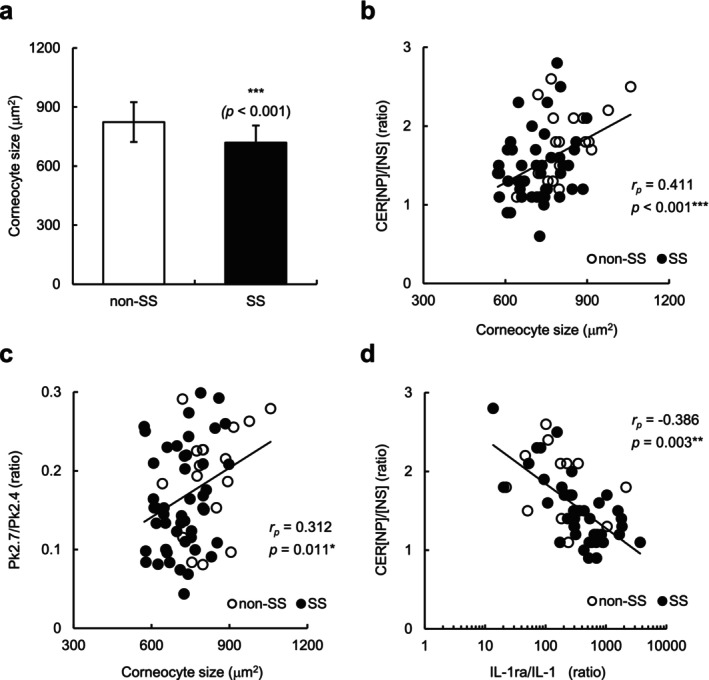
Corneocyte size in the SC of individuals with and without SS, and correlation between interleukin‐1 receptor antagonist (IL‐1ra)/IL‐1α and CER[NP]/[NS] ratios. (a) Corneocyte size in tape‐stripped SC from individuals without SS (non‐SS) (*n* = 18) and those with SS (*n* = 48). Values are expressed as mean ± SD (standard deviation). Statistical significance was assessed using Student's *t* test. ***p < 0.001. (b**–**c) Correlations between corneocyte size and CER[NP]/[NS] (b) and Pk2.7/Pk2.4 (c) ratios in all participants of the two groups (*n* = 66). (d) Correlation between IL‐1ra/IL‐1α and CER[NP]/[NS] ratios in tape‐stripped SC from participants of the two groups (*n* = 57). Open and closed circles indicate non‐SS and SS, respectively. Correlations were examined using Pearson's correlation analysis. *r*_*p*_, Pearson's correlation coefficient. ***p < 0.001; **p < 0.01; *p < 0.05.

To examine a possible relationship between the epidermal turnover rate and the altered CER[NP]/[NS] ratio, we assessed the maturation pattern of corneocytes by measuring the size of tape‐stripped corneocytes. As shown in Figure 4a, the corneocyte size in individuals with SS was significantly smaller than that in individuals without SS (*p* < 0.001), suggesting that individuals with SS had an increased epidermal turnover rate. Importantly, the corneocyte size was significantly correlated with the CER[NP]/[NS] ratio (*p* < 0.001) (Figure 4b) and Pk2.7/Pk2.4 ratio (*p* = 0.011) (Figure 4c) and tended to be correlated with the LAST score (*r*_s_ = −0.240, *p* = 0.053) (Table 3) in the participants. Because the corneocyte size is known to be reduced in inflamed skin, we evaluated the possible involvement of inflammation in the abnormal CER[NP]/[NS] ratio of individuals with SS by evaluating an indicator of chronic inflammation. Although no significant difference was found in the IL‐1ra/IL‐1α ratio in the SC between individuals with SS and those without SS (data not shown), the IL‐1ra/IL‐1α ratio was negatively correlated with the CER[NP]/[NS] ratio (*p* = 0.003) (Figure 4d). These results suggest that chronic low‐level inflammation may occur in individuals with SS, which in turn contributes to the abnormal CER[NP]/[NS] ratio, epidermal turnover rate, and lipid organization.

## 4 Discussion

Despite physiological similarities including skin hydration and redness between individuals with and without SS in this study, individuals with SS had a slight decline in facial skin barrier function and abnormal lateral packing of ICLs. Further, CER profiles of individuals with SS exhibited lower levels of CER[NH], [NP], [EOS], [EOH], and [EOP], and the CER[NP]/[NS] ratio was particularly correlated with the LAST score. Abnormal epidermal turnover may be a mechanism underlying the abnormalities observed in individuals with SS.

Previous studies have suggested a link between reduced content of total CERs and “self‐perceived” SS; however, the results remain controversial [5, 6]. In this study, no significant differences were found in the levels of total CERs, total ICLs, FFAs, free CHOL, and CHOL sulfate between individuals with SS and those without SS. Meanwhile, the levels of CER[NH], [NP], [EOS], [EOH], and [EOP], as well as the CER[NP]/[NS] ratio, were significantly or tended to be lower in individuals with SS. These data imply that the combined selection of self‐assessment questionnaires and LAST in this study may succeed in detecting true physiological differences in individuals with SS, rather than only psychological changes. One of the most interesting findings of this study was that the CER[NP]/[NS] ratio was directly correlated with the LAST score. In addition, the CER[NP]/[NS] ratio was the most greatly related to the lower orthorhombic ratio of ICLs among the levels of CERs examined. Previously, skin lipid model membranes, which mainly comprise hexagonal lateral packing, displayed higher permeability to ethyl‐*p*‐aminobenzoic acid as an increase in the free volume of the lipid membranes facilitated the intradermal transport of small molecules [15]. Therefore, the lower orthorhombic order of ICLs in individuals with SS, which is at least in part mediated by the altered CER[NP]/[NS] ratio, may contribute to easier penetration of external irritants into the skin.

To date, CER[NS] and CER [NP] have been demonstrated to be produced from CER[NDS] through the actions of dihydroceramide desaturases 1 and 2 (DES1 and DES2), respectively. During keratinocyte differentiation, DES2 expression is upregulated, whereas DES1 is constitutively expressed [16]. Thus, decreased DES2 expression caused by impaired keratinocyte cornification in SS may result in decreased CER[NP] production, thereby leading to decreased CER[NP]/[NS] and lower orthorhombic ratios. Actually, we obtained data showing that corneocyte size positively correlated with both ratios. Another interesting finding of this study was that the IL‐1ra/IL‐1α ratio, an indicator of chronic inflammation [17], was negatively correlated with the CER[NP]/[NS] ratio. Although individuals with SS did not show apparent signs of inflammation, IL‐1α‐mediated low‐level inflammation may have influenced the CER profile of SS. This notion is supported by a previous finding that treatment with IL‐1α decreased CER[NP] production in the SC of a reconstructed human epidermal model [18]. Therefore, chronic subclinical inflammation may occur in SS.

Previously, increases in short‐chain CERs, particularly CER[NS], were also involved in altered lipid organization and decreased barrier function in ad patients [3, 7]. However, the average carbon chain length of all CERs, including CER[NS], was not shortened in individuals with SS in the present study. Thus, this difference may explain the discrepancy in the severity of skin barrier function damage between ad and mild‐to‐moderate SS. Interestingly, a previous study suggested a link between CER[AS] or [AP] and TEWL in individuals between the ages of 25 and 69 years [19], but no significant correlations were found between them in this study. Although further analyses are needed, these differences may imply that the relationship between CER[AS] or [AP] and TEWL might be stronger in higher‐age individuals. Moreover, CER[NP]/[EOS] or [NP]/[AH] ratios did not remarkably correlate with the Pk2.7/Pk2.4 ratio and skin physiological data (data not shown).

To date, dry skin [20] and capsaicin‐sensitive skin [2] showed a greater number of smaller corneocytes, and the smaller surface area was considered to indicate lower maturity of corneocytes due to the accelerated epidermal turnover rate. In this study, corneocyte size was significantly smaller in individuals with SS than in non‐SS, suggesting inferior SC maturation in individuals with SS, although further direct analyses are needed to clarify the hypothesis. Interestingly, a previous study suggested that a smaller corneocyte size results in a reduced penetration pathway for external irritants [2]. Therefore, in addition to the lower orthorhombic ratio of ICLs, the reduction in corneocyte size may also contribute to an easier skin penetration rate in SS.

It has been reported that 20% of intrinsic ad (Iad) patients are positive for LAST [8, 21]. The clinical phenotype of ad patients is classified as extrinsic ad (EAD), which shows disrupted skin barrier function and high total IgE levels > 400 kU/L, and IAD, which has relatively preserved barrier function and normal total IgE levels ≤ 200 kU/L [21]. IAD accounts for approximately 20% of all AD cases and is predominantly observed in females. In the current study, individuals with SS were females and had a slightly impaired barrier function. In addition, the total IgE levels in individuals with SS were equivalent to those in IAD patients. These lines of observations suggest that individuals with SS share some similar pathophysiological properties with patients with IAD. Of note, based on the a* and EI values of the facial skin, individuals with SS lacked apparent visible signs of clinical inflammation. Therefore, although further studies are needed, one can speculate that some individuals with SS may, at least in part, have a subclinical form of IAD.

One limitation of this study was that we showed only correlations between SS and SC parameters, and therefore causal relationships between them need to be clarified. Moreover, because all the correlation coefficients were generally low, a possible involvement of covalently bound CER abnormalities in the hypersensitivity of SS is now under investigation in our group. Another potential limitation was that our study was performed only on the facial skin and included only young to middle‐aged Japanese females between the ages of 20 and 49 years. Therefore, further studies are desired to be conducted on different skin locations, females in different age groups, males, and/or individuals in different ethnic groups to generalize our conclusion.

In summary, we provided the first evidence that changes in the CER profile, particularly the CER[NP]/[NS] ratio, of individuals with mild‐to‐moderate SS who were characterized by no apparent dry skin nor erythema are linked to disordered ICL structure and skin hypersensitivity. Abnormal epidermal turnover may be the underlying mechanism of the altered CER[NP]/[NS] ratio through impaired keratinocyte cornification in SS. Therefore, in addition to a topical application of pseudo‐CER [22], a skincare strategy to intrinsically correct CER abnormalities may also be beneficial for healthy individuals with SS to ameliorate impaired barrier function and skin sensations.

## Author Contributions

T.J., H.Y., D.W., N.Y., A.K., and M.S. designed research; T.J., H.K., L.Z., D.W., and M.H. performed research; T.J., H.Y., H.K., L.Z., D.W., N.Y., M.H., S.N., A.K., and H.N. analyzed data; T.J., H.Y., and M.H. wrote the manuscript; T.J., H.Y., H.N. and M.S. reviewed and revised the manuscript. The authors approved the final manuscript.

## Ethics Statement

This study was conducted in accordance with the Declaration of Helsinki and was approved by the ethics committee of Kao Corporation, Japan (D164‐210115). Informed consent was obtained from all participants.

## Conflicts of Interest

The authors declare no conflicts of interest.

## Supporting information


Figures S1–S2.

## Data Availability

Research data are not shared.
