# Supplementary material for: Altered Ceramide Profile of Facial Sensitive Skin: Disordered Intercellular Lipid Structure Is Linked to Skin Hypersensitivity
Source: J Cosmet Dermatol. 2025 Apr 2;24(4):e70154. doi: 10.1111/jocd.70154 (PMC11965967; doi:10.1111/jocd.70154)
Supplement: Supplementary file 1 — Figures S1–S2. [file JOCD-24-e70154-s001.docx]

**Supporting Information**

**Figure S1. Average carbon chain length of ceramide (CER) subclass in the stratum corneum (SC) of individuals with and without sensitive skin (SS).**

Average carbon atoms of CER[NDS], [NS], [NH], [NP], [ADS], [AS], [AH], [AP], [EOS], [EOH], and [EOP] in tape-stripped SC from individuals without SS (non-SS) (n=18) and those with SS (n=48). Values are expressed as mean±SD (standard deviation). Statistical significances were assessed using Student’s *t* test. *, *p*<0.05. N.S., not significant.

**Figure S2. Free fatty acids (FFAs) in the SC of individuals with and without SS.**

(a-c) Levels of linear FFAs (a), branched FFAs (b), and unsaturated FFAs (c) containing 22-30 total carbon atoms in the tape-stripped SC from individuals without SS (non-SS) (n=18) and those with SS (n=48). Values are expressed as mean±SD (standard deviation). Statistical significances were assessed using Student’s *t* test. N.S., not significant.
